# cDNA Cloning and Partial Characterization of the *DJ-1* Gene from *Tribolium castaneum*

**DOI:** 10.3390/antiox10121970

**Published:** 2021-12-10

**Authors:** Shunya Sasaki, Maaya Nishiko, Takuma Sakamoto, Michael R. Kanost, Hiroko Tabunoki

**Affiliations:** 1 Department of Science of Biological Production, Graduate School of Agriculture, Tokyo University of Agriculture and Technology, Fuchu 183-8504, Tokyo, Japan; sasakislack@gmail.com (S.S.); tsakamoto@go.tuat.ac.jp (T.S.); 2 United Graduate School of Agricultural Science, Tokyo University of Agriculture and Technology, Fuchu 183-8504, Tokyo, Japan; n.maaya1222@gmail.com; 3 Institute of Global Innovation Research, Tokyo University of Agriculture and Technology, 3-5-8 Saiwai-cho, Fuchu 183-8509, Tokyo, Japan; 4 Department of Biochemistry and Molecular Biophysics, Kansas State University, 141 Chalmers Hall, Manhattan, KS 66506, USA; kanost@ksu.edu

**Keywords:** DJ-1, superoxide dismutase, oxidative stress, *Tribolium castaneum*, paraquat

## Abstract

The *DJ-1* gene is highly conserved across a wide variety of organisms and it plays a role in anti-oxidative stress mechanisms in cells. The red flour beetle, *Tribolium castaneum*, is widely used as a model insect species because it is easy to evaluate gene function in this species using RNA interference (RNAi). The *T. castaneum* *DJ-1* (*TcDJ-1*) sequence is annotated in the *T. castaneum* genome database; however, the function and characteristics of the TcDJ-1 gene have not been elucidated. Here, we investigated the cDNA sequence of TcDJ-1 and partially characterized its function. First, we examined the TcDJ-1 amino acid sequence and found that it was highly conserved with sequences from other species. *TcDJ-1* mRNA expression was higher in the early pupal and adult developmental stages. We evaluated oxidant tolerance in *TcDJ-1* knockdown adults using paraquat and found that adults with *TcDJ-1* knockdown exhibited increased sensitivity to paraquat. Our findings show that *TcDJ-1* has an antioxidant function, as observed for DJ-1 from other insects. Therefore, these results suggest that *TcDJ-1* protects against oxidative stress during metamorphosis.

## 1. Introduction

The DJ-1 gene was discovered to be a proto-oncogene in cultured mouse cells [1], and it has also been found to be a causative gene of Parkinson’s disease (PD), because mutations in the DJ-1 gene cause familial PD. PD is a neurodegenerative disease that affects movement by causing degeneration of dopaminergic neurons in the substantia nigra and by reducing the secretion of dopamine [2,3].

The DJ-1 gene is highly conserved across animal species and is proposed to play important roles related to antioxidant function [4]. The human DJ-1 protein is multifunctional and plays roles in antioxidant ability, fertility, and protease activity [5,6,7,8]. DJ-1 is activated by oxidative modification and is rapidly oxidized at position Cys 106 [9]. One function of DJ-1 is to protect cells from oxidative damage to the mitochondria. Oxidative modification leads to mitochondrial damage in cultured cells exposed to several compounds, such as paraquat, which inhibits the electron transport chain of mitochondrial complex I [10]. Paraquat enhances the production of reactive oxygen species (ROS) and reduces the production of ATP, resulting in mitochondrial dysfunction [11]. Therefore, DJ-1 seems to directly scavenge free radicals from mitochondria in response to oxidative stress.

Insect *DJ-1* function has been analyzed in two species, *Drosophila melanogaster* and *Bombyx mori*. *D. melanogaster* has two DJ-1 genes; *DJ-1alpha* (DmDJ-1α Gene ID: 36543) and *DJ-1 beta* (DmDJ-1β Gene ID: 43652). *DJ-1 alpha* is located on chromosome 2R, whereas *DJ-1 beta* is located on chromosome 3R. Additionally, *DJ-1beta* is expressed in the whole body, but DmDJ-1α is mainly expressed in the testis. Therefore, they play different roles in controlling oxidative stress [12]. Flies with double knockdown of DmDJ-1α and DmDJ-1β have increased sensitivity to oxidative stress from H_2_O_2_ and paraquat. Therefore, *DmDJ-1s* have antioxidant functions which are similar to those of mammalian *DJ-1* [13]. We also observed that knockout of *D. melanogaster DJ-1* resulted in a shortened lifespan and abnormal spermatogenesis [13].

The expression of the *B. mori* DJ-1 (BmDJ-1) protein fluctuates depending on the concentration of nitric oxide (NO) in the hemolymph. BmDJ-1 might play a role in the regulation of NO generated during molting and metamorphosis [14]. Therefore, the function of insect DJ-1 is similar to that of human DJ-1 with regard to resistance to oxidative stress, and insect DJ-1 is expected to be involved in the regulation of biological events related to ROS.

The red flour beetle (*Tribolium castaneum*), a coleopteran insect, is known as a pest of wheat flour and other stored food products. The whole genome of *T. castaneum* has been elucidated [15] and the insect is easy to maintain and is highly fertile. In addition, RNA interference (RNAi) by injection of double-stranded RNA (dsRNA) has remarkable effects on all developmental stages of *T. castaneum* [16,17]. Thus, *T. castaneum* is suitable for analysis of gene function by reverse genetic methods.

One *T. castaneum* DJ-1 (TcDJ-1) sequence is annotated in the *T. castaneum* genome database; however, the function and characteristics of TcDJ-1 have not been elucidated. In this study, we investigated cDNA sequence and partial characterization of TcDJ-1 lack of function by RNAi. We also analyzed the relationships of TcDJ-1 and other anti-oxidative stress genes, superoxide dismutases, in metamorphosis.

## 2. Materials and Methods

### 2.1. Insects

The *T. castaneum* GA-1 strain was used in all experiments. The insects were reared on whole wheat flour containing 5% brewer’s yeast and maintained at 27 °C under a 16-h light/8 h dark cycle. The *B.mori* p50T strain was gifted by Dr. Katsuhiko Ito (Tokyo University of Agriculture and Technology, Tokyo, Japan). Larvae were reared on an artificial diet of Silkmate 2S (NOSAN, Tsukuba, Japan) and maintained at 25 °C under a 16 h light/8 h dark cycle. The *D. melanogaster* Canton-S strain was gifted by Dr. Tsunaki Asano (Tokyo Metropolitan University, Tokyo, Japan). The larvae were reared on the Drosophila Diet Medium K12, High Efficiency (U.S. Biological, Salem, MA, USA) including 0.23% Tegosept (U.S. Biological) and maintained at 25 °C under a 16 h light/8 h dark cycle. 

### 2.2. Identification of the TcDJ-1 Sequence and Bioinformatics Analysis

A search for *T. castaneum* DJ-1 and DJ-1 orthologues in other organisms was performed with the NCBI database (https://www.ncbi.nlm.nih.gov/protein, accessed on 7 July 2018). Global homology searches were conducted using Genetyx ver. 11 (Genetyx Co. Ltd., Tokyo, Japan). Alignment of the deduced *TcDJ-1* amino acid sequences with DJ-1 homologs from other species (Supplementary Materials Table S1) was conducted using the Genetyx ver. 11, protein vs. protein homology program. Phylogenetic analysis was then conducted using the MEGA X program [18]. A protein motif search was conducted using InterProScan (https://www.ebi.ac.uk/interpro/search/sequence-search, accessed on 3 July 2018). 

### 2.3. Immunoblotting

To examine the differences between the TcDJ-1 and BmDJ-1 protein structures, we performed immunoblotting using *B.mori* DJ-1 rabbit-antiserum [14]. Midguts were dissected from *T. castaneum* old larvae, *B.mori* fifth instar larvae, and *D. melanogaster* final instar larvae using 1% BSA containing PBS pH6.5. These midguts were weighed and homogenized with three volumes of protein lysis buffer (ATTO Co. Ltd., Tokyo, Japan), including a protease inhibitor cocktail (Sigma St. Louis, MO, USA). The protein concentration was determined by a Bradford assay kit (Pierce, Rockford, IL, USA). Protein samples (100 μg) were separated on SDS-PAGE, transferred to nitrocellulose membranes using the method of Towbin et al. [19], and immunoblotted using rabbit-antiserum against BmDJ-1 and goat anti-rabbit IgG-conjugated horseradish peroxidase (HRP). The membranes were developed using a chemiluminescent substrate (BIO-RAD, Hercules, CA, USA), and the signals were visualized using WSE-6200HLuminoGraphII (ATTO Co. Ltd.).

### 2.4. Total RNA Purification from Whole-Body Samples and cDNA Synthesis

Each whole body (*n* = 3) was homogenized with TRIzol™ Reagent (Life Technologies, Carlsbad, CA, USA) and processed for total RNA purification in accordance with the manufacturer’s instructions. Three replications of total RNA from each developmental stage were stored at −80 °C until use. Total RNA (1 μg) was treated with amplification-grade deoxyribonuclease (DNase) I (Life Technologies). Afterward, 500 ng of DNase-treated total RNA was used as a template for cDNA synthesis using a PrimeScript™ 1st Strand cDNA Synthesis Kit (Takara Bio, Inc., Kusatsu, Shiga, Japan). cDNA cloning was performed using specific primers (Supplementary Materials, Table S2). The amplified products were cloned using a TOPO^®^ TA Cloning^®^ Kit for Subcloning (Thermo Fisher Scientific, Waltham, MA, USA) and cloned with Competent Quick XL-1 blue cells (TOYOBO Co. Ltd., Tokyo, Japan). The purified vectors were processed for sequencing using an Applied Biosystems 3730xl DNA analyzer.

### 2.5. Quantitative Real-Time PCR (qRT-PCR)

qRT-PCR was performed in 20 μL reaction volumes, consisting of 0.125 μL of the cDNA template and specific primers (Table S3) with a KAPA SYBR^®^ FAST qPCR Kit Master Mix (2X) ABI Prism™ (Sigma-Aldrich Corporation, St. Louis, MO, USA), in accordance with the manufacturer’s instructions, and with a StepOnePlus™ Real-Time PCR System (Applied Biosystems, Carlsbad, CA, USA). Relative gene expression was calculated using the 2^–∆∆Ct^ method with the *T. castaneum* ribosomal protein S6 gene (*RpS6*, gene identification (ID) number 288869507) as an endogenous reference for standardization of RNA expression levels. All data were calibrated against universal reference data. The relative expression levels compared to the levels in a reference sample are presented as the relative quantification (RQ) values. All samples were assayed in three technical replicates and checked in three biological replicants.

### 2.6. Synthesis and Injection of dsRNA

The online tool, E-RNAi version 3.2 [20] (https://www.dkfz.de/signalling/e-rnai3/evaluation.php, accessed on 11 January 2019), was used to evaluate possible off-target effects of dsRNA. *TcDJ-1* dsRNA was synthesized from 310 bp of the target site (base pairs 251–561 in the open reading frame (ORF)) and amplified by RT-PCR using the *T. castaneum* larval cDNA library. The primers used for RT-PCR amplification are listed in Table S2. The resulting fragment was ligated into a vector using a TOPO^®^ TA Cloning^®^ Kit for Subcloning (Thermo Fisher Scientific), cloned with Competent Quick XL-1 blue cells (TOYOBO Co., Ltd.) and sequenced. *T. castaneum vermillion* (*TcVer*, Gene ID; 288869507) was used as a negative control. The dsRNA for each target was synthesized with a T7 RiboMAX™ Express RNAi System (Promega Corporation, Madison, WI, USA) in accordance with the manufacturer’s protocols. Old *T. castaneum* larvae were injected with 400 ng/200 nL of dsRNA using a microinjection system (Narishige Co. Ltd., Tokyo, Japan) under a stereomicroscope. A qRT-PCR of 5-day-old pupae was performed at 8 days after dsRNA injection. A qRT-PCR was performed with the specific primers listed in Table S3 to assess the knockdown efficiency of the target genes. In addition, the phenotype of each insect group was investigated. Adult survivorship was calculated as the number of live insects over a period of 61 days starting from adult day 0.

### 2.7. Determination of the LC50 of Paraquat in TcDJ-1 Knockdown Adults

The LC50 (the concentration at which half of the treated individuals were killed) of paraquat in *TcDJ-1* knockdown adults was examined according to the methods of Tabunoki et al. [21]. Paraquat was administered to *TcVer* knockdown adults (*n* = 10 for each paraquat concentration) and *TcDJ-1* knockdown adults (*n* = 5 for each paraquat concentration) one month after treatment with *TcVer* or *TcDJ-1* dsRNA. The number of dead insects after 24 h was counted, and the mortality rate was calculated as follows: mortality rate = (*X*/*Y*), where *X* = the number of dead insects in the group and *Y* = the total number of insects in the group (a value of 1 indicates 100% survival). The LC50 values were calculated by using the probit analysis [22] option in the JMP 10.0 software package (SAS Institute Japan Ltd., Tokyo, Japan).

### 2.8. Examination of the Relationship of TcDJ-1 with the Antioxidant Proteins, Superoxide Dismutases (SODs)

*T. castaneum* prepupae (*n* = 3) were injected with 400 ng/200 nL *TcVer* or *TcDJ-1* dsRNA using a microinjection system (Narishige Co. Ltd.) under a stereomicroscope. The samples were then collected at days 0–6 of the pupal stage. Afterward, qRT-PCR was conducted using specific primers, as listed in Table S3, to assess the knockdown efficiency of target genes. Additionally, the phenotype of each insect group was investigated.

## 3. Results

### 3.1. Identification and Characterization of the TcDJ-1 Nucleotide Sequence

We obtained the *TcDJ-1* nucleotide sequence from the NCBI database. We obtained the *TcDJ-1* nucleotide sequence from the NCBI database. One reference sequence was found from the NCBI database (XM_968208). This model sequence was annotated as *T. castaneum DJ-1* (TC014965) and was localized at LG6:5823597-5824695. Next, we amplified the *TcDJ-1* cDNA sequence through PCR with specific primers, after which we confirmed the nucleotide sequence of *TcDJ-1* through cDNA cloning and sequencing. Searching of the *T. castaneum* genome with BLAST did not reveal any other *TcDJ-1* homologs. The nucleotide sequence reported in this paper has been submitted to the DDBJ Annotated/Assembled Sequences database under Accession No. LC628081. The deduced ORF was 561 nucleotides long and encoded a protein of 186 amino acids, with a molecular weight of 19314.09 Da and a putative isoelectric point of 6.94. *TcDJ-1* contained a DJ-1/Pfp I domain (DJ-1/Pfp I, Pfam; PF01965) at position 4-167. *TcDJ-1* was very similar in sequence to homologs from other species, including *B. mori DJ-1* (87% similarity), *D. melanogaster DJ-1* (alpha, 82%; beta, 88%), *Anoplophora glabripennis* DJ-1 (X1, 92%; X2, 92%), and human *DJ-1* (83%) (Table S4). In the phylogenetic analysis of *TcDJ-1*, clusters of insect species were separated by order (Supplementary Materials, Figure S1). *TcDJ-1* belonged to the Coleoptera cluster (Supplementary Materials, Figure S1). Of the three cysteine residues involved in the anti-oxidative stress function of *human DJ-1*, those at positions 46 and 106 were conserved in *TcDJ-1*. In contrast, the cysteine residue at position 53 was replaced by a lysine residue in *T. castaneum* (Table 1). The histidine at position 126, which is involved in the protease activity of intracellular protease PH1704 and E. coli heat shock protein 31 [7,23,24], was replaced with tyrosine in *TcDJ-1* (Table 1).

### 3.2. Examination of Cross-Reactivity in TcDJ-1, and BmDJ-1

**Figure 1:**
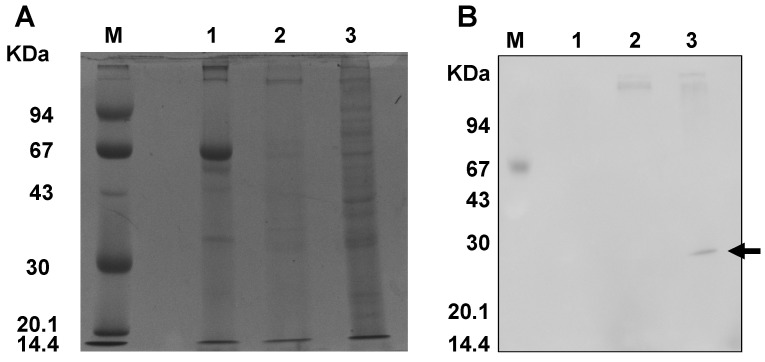
Cross-reactivity of TcDJ-1 and BmDJ-1 proteins. Protein lysates (100 μg) were separated on a 12% SDS-PAGE gel, transferred onto a nitrocellulose membrane, and processed for immunoblotting with BmDJ-1 antibody. The following samples were loaded in each lane; M, marker; 2, *T. castaneum* midgut; 3, *D. melanogaster* midgut as a negative control; 4, *B. mori* midgut as a positive control. (**A**) SDS-PAGE, (**B**) immunoblotting.

To examine the differences between TcDJ-1 and BmDJ-1 protein structures, we performed immunoblotting using BmDJ-1 rabbit-antiserum. The BmDJ-1 antiserum did not react with TcDJ-1 proteins (Figure 1).

### 3.3. Examination of TcDJ-1 mRNA Expression during Development

**Figure 2:**
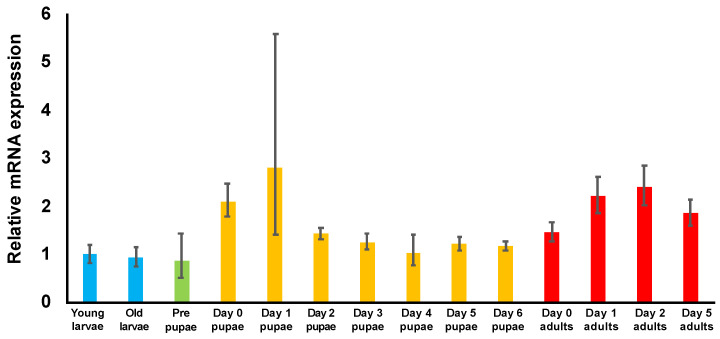
Expression of *TcDJ-1* in *T. castaneum* at different developmental stages. Whole bodies of beetles at each developmental stage were used for qRT-PCR. The expression level of *TcDJ-1* mRNA is shown compared to the expression level in young larvae, which was set as 1. The expression levels of larvae, pharate pupae, pupae, and adults are shown as RQ values. The RQ represents the relative expression level compared to the reference sample. The error bars represent the relative minimum/maximum expression levels relative to the mean RQ value. *TcRpS6* was used as an endogenous control. Relative expression levels were calculated using young larvae samples as the reference.

To examine *TcDJ-1* mRNA expression levels in *T. castaneum*, from the young larval stage to the 5-day adult developmental stage, we performed qRT-PCR analysis. The expression of *TcDJ-1* mRNA was higher in the early pupal and adult developmental stages than in the young larval stage (Figure 2). Additionally, *TcDJ-1* mRNA expression had a similar pattern between males and females (Figure S2A,B). Therefore, *TcDJ-1* mRNA expression was increased after pupation and adult eclosion.

### 3.4. Evaluation of TcDJ-1 Knockdown Efficiency

**Figure 3:**
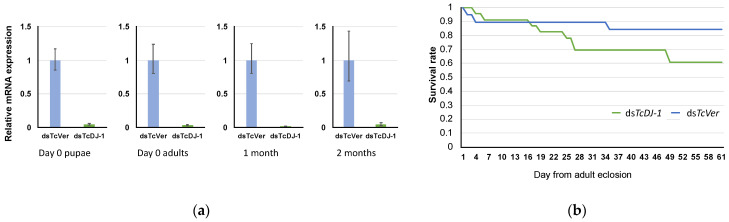
(**a**) Verification of *TcDJ-1* knockdown efficiency. The time course of *TcDJ-1* mRNA expression was examined by qRT-PCR. The expression level of *TcDJ-1* mRNA is shown compared to the expression level in the *TcVer* knockdown control group, which was set as 1. *TcRpS6* was used as an endogenous control. (**b**) Effect of *TcDJ-1* knockdown on the adult life span of *T. castaneum*. Prepupae were treated with *TcDJ-1* or *TcVer* dsRNA. The live insects were counted for 61 days beginning on adult day 0. Each value shows the rate of survival (1 = 100% survival) for each group.

The knockdown efficiency of *TcDJ-1* dsRNA was verified by qRT-PCR. The expression of *TcDJ-1* mRNA was lower in the *TcDJ-1* dsRNA-injected groups than in the *TcVer* dsRNA-injected control groups (Figure 3a). Knockdown via *TcDJ-1* dsRNA injection was maintained for at least two months.

Next, we observed the survival of *T. castaneum* adults for 61 days and found that the *TcDJ-1* knockdown group had a shorter lifetime than the *TcVer* control group (Figure 3b). However, statistical analysis did not show the differences after 61 days between the *TcDJ-1* and *TcVer* knockdown groups (*p* = 0.1).

### 3.5. Examination of the Relationship between TcSOD Expression and TcDJ-1 Knockdown

**Figure 4:**
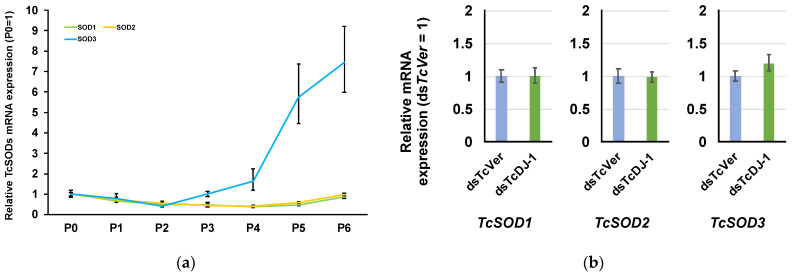
Examination of the expression of *TcSOD1*, *TcSOD2*, and *TcSOD3* mRNA in *TcDJ-1* knockdown individuals. (**a**) *TcSOD1*, *TcSOD2*, and *TcSOD3* mRNA expression during the pupal developmental stage. Relative expression levels were calculated using values obtained on day 0 of pupa sampling as the reference. (**b**) *TcSOD1*, *TcSOD2*, and *TcSOD3* mRNA expression was examined by qRT-PCR on day 6 in TcDJ-1 knockdown pupae. The expression level of *TcDJ-1* mRNA is shown compared to the expression level in the *TcVer* knockdown control group, which was set as 1. *TcRpS6* was used as an endogenous control.

SOD is also an important anti-oxidative stress protein that converts the superoxide anion to hydrogen peroxide via dismutase activity [25]. Three types of *SOD* genes (*TcSOD1*, *TcSOD2*, and *TcSOD3*) have been reported in *T. castaneum* [21,26,27]. These SODs play a role in metamorphosis. *TcDJ-1* mRNA expression was also increased during metamorphosis. Thus, we examined the relationships of *TcSOD3*, *TcSOD1*, and *TcSOD2* mRNA in TcDJ-1 knockdown pupae. First, we examined the mRNA expression of *TcSODs* during the pupal developmental stage. We found that the *TcSOD3* mRNA level was higher on day six and lower on day two of pupal development than on day 0. However, *TcSOD1* and *TcSOD2* were low throughout the pupal developmental stages (Figure 4a). Thus, we used day 6 of pupal development to evaluate the relationships between TcDJ-1 and *TcSOD3* in this study. The expression levels of *TcSOD3* mRNA in 6th-day pupae were compared between the *TcDJ-1* dsRNA-injected and TcVer dsRNA-injected groups. There was no difference in the expression level of *TcSOD3* mRNA between the two knockdown groups (Figure 4b). In addition, the expression of *TcSOD1* and *TcSOD2* mRNA did not fluctuate in the TcDJ-1 knockdown group (Figure 4b).

### 3.6. Examination of Paraquat-Induced Oxidative Stress in TcDJ-1 Knockdown Beetles

To assess oxidative stress resistance ability, we used paraquat to induce oxidative stress in the *TcDJ-1* knockdown adults and *TcVer* knockdown controls and found that the LC50 of the *TcVer* knockdown control group was 9.89 mM (95% CI = 3.66–23.96), whereas the LC50 of the *TcDJ-1* knockdown group was 1.56 mM (95% CI = lower than 7.58). Thus, the *TcDJ-1* knockdown group was slightly susceptible to paraquat-induced oxidative stress than the *TcVer* control group.

## 4. Discussion

In this study, we examined the sequence of *TcDJ-1* and partially characterized its function by observing phenotypes after knockdown by RNAi. We also analyzed the relationships of TcDJ-1 and other anti-oxidative stress genes, *TcSOD*s, in metamorphosis. The TcDJ-1 amino acid sequence was highly conserved with the sequences of other species. Human DJ-1 has three cysteine residues at positions 46, 53, and 106, which are oxidized to remove ROS when organisms are subjected to oxidative stress [28]. The cysteine residue at position 106 was conserved among all insect species examined in this study and is particularly important for the antioxidant function of the human DJ-1 protein. If these three cysteine residues are excessively oxidized, human DJ-1 protein cannot retain its conformation and its antioxidant function is disrupted [29,30]. In *TcDJ-1*, the residue corresponding to the cysteine at position 53 was replaced with a lysine residue (Table 1). The cysteine at position 53 was replaced with a valine residue in *B. mori DJ-1* (*BmDJ-1*), whereas the cysteine at position 53 was replaced with a leucine residue in *D. melanogaster DJ-1 beta* (*DmDJ-1 beta*). Nevertheless, the antioxidant function was conserved in both *BmDJ-1* and *DmDJ-1 beta* [12,13,14]. Knockdown of *TcDJ-1* decreased the antioxidant function in this study. Thus, the cysteine residues at positions 46 and 106 are proposed to be potentially important for the antioxidant function of *TcDJ-1*, whereas the amino acid residue at position 53 was less conserved and is proposed to not be required for antioxidant activity.

To examine the differences between TcDJ-1 and BmDJ-1 protein structures, we performed immunoblotting using BmDJ-1 rabbit-antiserum. Although TcDJ-1 has high homology with BmDJ-1, BmDJ-1 rabbit-antiserum did not react with the TcDJ-1 protein, as shown in Figure 1. Generally, cross-reactivity for a protein structure occurs when a polyclonal antibody recognizes similar structural regions; hence, our result suggests that TcDJ-1 has different structural regions which are not recognized by the BmDJ-1 rabbit-antiserum.

It has been reported that human DJ-1 protein exists as a dimer [31], and the leucine residue at position 166 was important for dimer formation as substitution of this residue prevents dimer formation [28,32]. The leucine residue at position 166 was conserved in the DmDJ-1 beta protein. Thereby it potentially also has a function in forming a dimer (https://www.rcsb.org/structure/4E08, accessed on 27 November 2021). TcDJ-1 is also conserved at this leucine residue, like DmDJ-1 beta. Therefore, the TcDJ-1 protein may be proposed to influence dimer formation.

Pfp I family proteins, which are bacterial intracellular proteases [33,34,35], and heat shock protein 31 (Hsp31) [6,24], which is an *Escherichia coli* chaperone, act as proteases. These proteins were found to be similar with DJ-1 family proteins in the current study. The DJ-1/Pfp I domain structure was also conserved in *TcDJ-1*. Crystal structure analysis of the human DJ-1 protein has shown that DJ-1 has a helix-strand-helix sandwich structure, similar to the structures of the bacterial proteases PH1704 and Hsp31 [23,24]. However, human DJ-1 does not have a Cys-His-Glu/Asp catalytic triad like those of PH1704 and Hsp31 [35]. Human DJ-1 has only a Cys-His diad, which could be responsible for protease function [36,37]. Notably, the Cys-His diad has a protease function in caspases [38,39,40]. We examined TcDJ-1 gene-encoded amino acid residues corresponding to the Cys-His diad at positions 106 and 126 and found that the histidine at position 126 was replaced with tyrosine in TcDJ-1 (Table 1). Thus, *TcDJ-1* probably lacks protease activity because of the substitution of tyrosine for an essential histidine to form a catalytic diad with the cysteine residue. Our examined insect DJ-1 proteins do not have the Cys-His diad for relation of protease activity, except for *Drosophila* DJ-1 alpha (Table 1).

Examination of *TcDJ-1* mRNA expression, from the larval through the adult developmental stages, revealed that *TcDJ-1* mRNA was most highly expressed in the early pupal and adult stages. Variation in *TcDJ-1* mRNA expression was highest on the first day of pupa development. However, the mean expression of *TcDJ-1* mRNA appeared to increase significantly from day 0 to day 1 of the pupal developmental stages. Similarly, *DJ-1β* (*DmDJ-1β*) mRNA expression in pupae and adults is approximately 2–3 times higher than that in larvae [41].

Metamorphosis occurs during the pupal developmental stage in holometabolous insects with remodeling of the body structure [42]. During this process, the level of reactive oxygen species is increased, thereby inducing apoptosis and autophagy for subsequent lysis of the larval body [43]. Our finding suggests that TcDJ-1 may play a role in protection from oxidative damage during metamorphosis, like DmDJ-1β.

*Human DJ-1* plays a role as an important regulator of antioxidant genes [44]. In mouse NIH3T3 cells, *DJ-1* knockdown causes a decrease in the expression of *SOD3* mRNA but does not affect the mRNA expression of *SOD1* and *SOD2* [28]. Insect SOD1 is located in the cytosol, SOD2 is located in mitochondria, and SOD3 is secreted to the hemolymph [45]. Thus, we examined the relationships of *TcDJ-1* and *Tribolium castaneum SOD3* (*TcSOD3*) in this study. *Tribolium castaneum SOD1*(*TcSOD1*), *TcSOD2*, and *TcSOD3* mRNA expression were not affected on day 6 of the pupa in the *TcDJ-1* knockdown group.

However, *TcSOD1* and *TcSOD2* mRNA expression was lower than *TcSOD3* in the pupal developmental stage (Figure 4a). Unlike the murine gene, *TcDJ-1* did not affect *TcSOD3* expression.

Paraquat is a herbicide that produces ROS by inhibiting the function of mitochondrial complex I [46] and induces oxidative stress in the cytosol [47]. Oxidative damage associated with the generation of ROS is thought to inhibit the proliferation of nerve cells and it has been used to create PD models in mammals and insects [48,49,50]. The LC50 value for orally administered paraquat was lower in the *TcDJ-1* knockdown group than in the *TcVer* knockdown group, and the survival rate in adults was also slightly reduced in the *TcDJ-1* knockdown group. DJ-1 is involved in protection from paraquat toxicity [51,52,53], and DmDJ-1β mutant flies have a significantly lower survival rate than controls after the administration of paraquat [13]. In this experiment, the decrease in the LC50 value suggests that the suppression of *TcDJ-1* mRNA slightly increased the sensitivity to paraquat. *TcDJ-1* knockdown beetles did not have responses similar to those of *DmDJ-1β* mutant flies. Nevertheless, our results showed that *TcDJ-1* also had an antioxidant effect on ROS.

## 5. Conclusions

In this study, we found that *TcDJ-1* likely has an antioxidant function similar to that of DJ-1 in *D. melanogaster* and *B. mori* DJ-1 and that *TcDJ-1* knockdown does not affect *TcSOD* mRNA expression during metamorphosis. *TcDJ-1* and *TcSODs* might have different roles in *T. castaneum* during metamorphosis. *TcDJ-1* is proposed to influence the removal of oxidative stress during metamorphosis. In future studies, we need to investigate the function of *TcDJ-1* at the protein level.

## Figures and Tables

**Table 1:** The functional amino acid residue in the DJ-1 orthologs.

Scientific Name	46	53	106	126
***Tribolium castaneum***	C	K	C	Y
***Agrilus planipennis***	C	K	C	Y
***Anoplophora glabripennis*** **X1**	C	K	C	Y
***Anoplophora glabripennis*** **X2**	C	N	C	Y
***Onthophagus taurus***	C	K	C	Y
***Dufourea novaeangliae***	C	C	C	Y
***Camponotus floridanus***	C	C	C	Y
***Melipona quadrifasciata***	C	C	C	Y
***Harpegnathos saltator***	C	C	C	Y
***Ooceraea biroi***	C	C	C	Y
***Trachymyrmex cornetzi***	C	C	C	Y
***Drosophila melanogaster* alpha**	C	V	C	H
***Drosophila melanogaster* beta**	C	L	C	Y
***Bactrocera latifrons***	C	V	C	Y
***Bactrocera dorsalis***	C	V	C	Y
***Papilio xuthus***	C	V	C	Y
***Papilio machaon***	C	V	C	Y
***Bombyx mori***	C	V	C	Y
***Homo sapiens***	C	C	C	H
***Mus musculus***	C	C	C	H
***Moesziomyces antarcticus***	C	V	C	H

Amino acid residue are shown as one character according to a one-letter notation for amino acid.

## Data Availability

The nucleotide sequence reported in this paper has been submitted to the DDBJ Annotated/Assembled Sequences database under Accession No. LC628081.

## Supplementary Materials

The following are available online at https://www.mdpi.com/article/10.3390/antiox10121970/s1: Figure S1: Phylogenetic tree of *T. castaneum* DJ-1 and other DJ-1 amino acid sequences.; Figure S2: Expression of *TcDJ-1* in *T. castaneum* males (A) and females (B) at different developmental stages. Table S1: Gene IDs used to create the phylogenetic tree in this study, Table S2: *TcDJ-1* primers used in cDNA cloning and dsRNA synthesis, Table S3: Primers used for qRT-PCR experiments, Table S4: Homology to sequences of other species.
