# Supplementary material for: cDNA Cloning and Partial Characterization of the DJ-1 Gene from Tribolium castaneum
Source: Antioxidants (Basel). 2021 Dec 10;10(12):1970. doi: 10.3390/antiox10121970 (PMC8750249; doi:10.3390/antiox10121970)
Supplement: Supplementary file 1 [file antioxidants-10-01970-s001.zip › antioxidants-1476813-supplementary.pdf]

Supplementary information

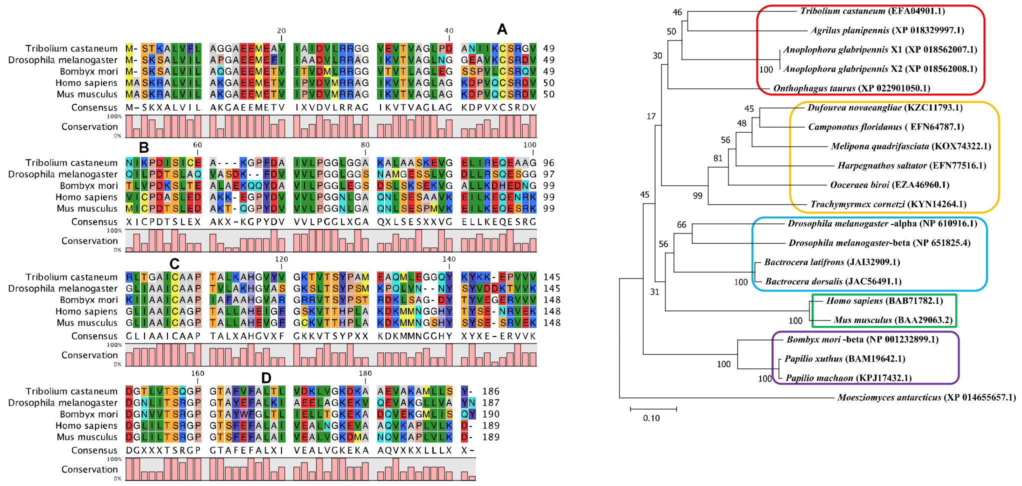

(a) (b)

Figure S1. Alignment and the phylogenetic tree of *T. castaneum* DJ-1 and other DJ-1 amino acid sequences. (a) alignment of DJ-1 homologs. A, cysteine 46; B, cysteine 53; C, cysteine 106; D, leucine 166. Each amino acid position corresponds to human DJ-1. (b) Phylogenetic tree. The Coleoptera cluster is outlined in red, the Hymenoptera cluster is outlined in yellow, the Diptera cluster is outlined in light blue, and the Lepidoptera cluster is outlined in purple. The numbers above the branches represent the recall rate of each branch as a percentage when the bootstrap test was conducted 1000 times, following the neighbor-joining method.

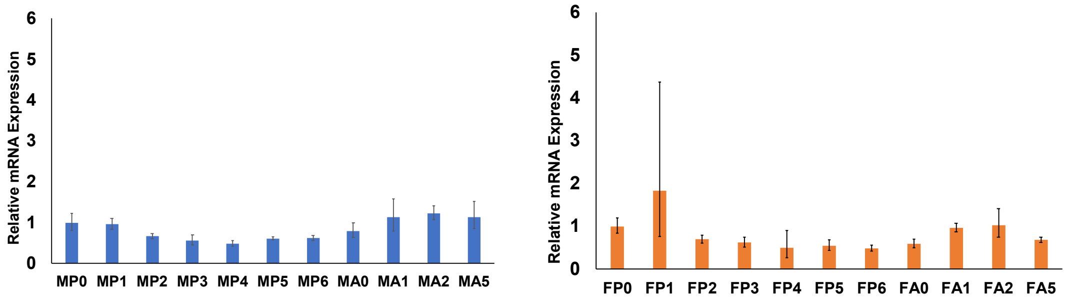

(**a**) (**b**)

Figure S2. Expression of TcDJ-1 in *T. castaneum* males (a) and females (b) at different developmental stages. Whole bodies of insects at each developmental stage were used for qRT-PCR. The expression level of *TcDJ-1* mRNA is shown compared to the expression level in day 0 pupae, which was set as 1. The expression levels of pupae, and adults are shown as RQ values. The RQ represents the relative expression level compared to the reference sample. The error bars represent the relative minimum/maximum expression levels relative to the mean RQ value. *TcRpS6* was used as an endogenous control.

Table S1. Accession numbers were used the alignment and phylogenic tree in this study.

Accession number	Scientific name
LC628081	*Tribolium castaneum*
XP_018329997.1	*Agrilus planipennis*
XP_018562007.1	*Anoplophora glabripennis X1*
XP_018562008.1	*Anoplophora glabripennis X2*
XP_022901050.1	*Onthophagus taurus*
KZC11793.1	*Dufourea novaeangliae*
EFN64787.1	*Camponotus floridas*
KOX74322.1	*Melipona quadrifasciata*
EFN77516.1	*Harpegnathos saltator*
EZA46960.1	*Ooceraea biroi*
KYN14264.1	*Trachymyrmex cornetzi*
NP_610916.1	*Drosophila melanogaster alpha*
NP_651825.4	*Drosophila melanogaster beta*
JAI32909.1	*Bactrocera latifrons*
JAC56491.1	*Bactrocera dorsalis*

BAM19642.1	*Papilio xutus*
KPJ17432.1	*Papilio machaon*
NP_001232899.1	*Bombix mori*
NP_001116849.1	*Homo sapiens*
NP_065594.2	*Mus Musculus*
XP_014655657.1	*Moesziomyces antarcticus*

Table S2 Primers used in cDNA cloning and dsRNA synthesis

TcVer for cDNA clonning	Forward	5’-TGATCCTCAAGGTATTGGTG-3’
	Reverse	5’-TGATCAGCGAGTCTATGTCC-3’
TcVer+T7	Forward	5'-TAATACGACTCACTATAGGTGATCCTCAAGGTCTTGGTG-3'
	Reverse	5'-TAATACGACTCACTATAGGTGATCAGCGAGTCTATGTCC-3'
TcDJ-1 for cDNA clonning	Forward	5'-ATGAGCACAAAAGCCCTTGT-3'
	Reverse	5'-TTAATACGACAAAAGCATGGCTT-3'
TcDJ-1+T7	Forward	5'-TAATACGACTCACTATAGGGAAGTAGGGGAGTTGAT-3'
	Reverse	5'-TAATACGACTCACTATAGGGTTAATACGACAAAAGCATG-3'

Table S3. Primers used for qRT-PCR experiments

TcRpS6	Forward	5’-GGACCCAAAAGAGCATCAAA-3’
	Reverse	5’-CCTCAAGCAACGCTTCTTCT-3’
TcVer	Forward	5'-CGTAATGAGTTGCCCACTGAGAC-3'
	Reverse	5'-CGACTCTTCTAAAACGTCGCTGA-3'
TcDJ-1	Forward	5'-GCAGAGGCGGTGTGGAAGTA-3'
	Reverse	5'-GGGCCTTTGGCCTCACAGAT-3'
TcSOD1	Forward	5'-GGTGATGGAGTTGCCAAAGT-3'
	Reverse	5'-GATTCCAACAACACCACACG-3'
TcSOD2	Forward	5'-CAAGTCCGGGAAATTGAAAA-3'
	Reverse	5'-CTCGAAAATGGCCTTGACAT-3'
TcSOD3	Forward	5'-CTTCCACATCCACGAGAAGG-3'
	Reverse	5'-CACCACAGCCCTCCCTATAA-3'

Table S4. Homology to the other species

Species	Identity %	Similarity %

Agrilus planipennis	60	91
Anoplophora glabripennis X1	64	92
Anoplophora glabripennis X2	64	92
Onthophagus taurus	61	89
Dufourea novaeangliae	51	89
Camponotus floridanus	53	88
Melipona quadrifasciata	52	89
Harpegnathos saltator	47	87
Ooceraea biroi	51	91
Trachymyrmex cornetzi	52	89
Drosophila melanogaster alpha	54	82
Drosophila melanogaster beta	54	88
Bactrocera latifrons	60	86
Bactrocera dorsalis	59	87
Papilio xuthus	50	86
Papilio machaon	50	86
Bombyx mori	52	87
Homo sapiens	49	83
Mus musculus	50	84
